# Development of a survey instrument to measure patient experience of integrated care

**DOI:** 10.1186/s12913-016-1437-z

**Published:** 2016-06-01

**Authors:** Kara Odom Walker, Anita L. Stewart, Kevin Grumbach

**Affiliations:** Patient Centered Outcomes Research Institute, Washington, DC USA; Institute for Health & Aging, Center for Aging in Diverse Communities, University of California-San Francisco, San Francisco, CA USA; Department of Family and Community Medicine, University of California-San Francisco, San Francisco, CA USA; 1828L Street NW, Washington, DC USA

**Keywords:** Scale development, Integrated care, Patient experience

## Abstract

**Background:**

Healthcare systems are working to move towards more integrated, patient-centered care. This study describes the development and testing of a multidimensional self-report measure of patients’ experiences of integrated care.

**Methods:**

Random-digit-dial telephone survey in 2012 of 317 adults aged 40 years or older in the San Francisco region who had used healthcare at least twice in the past 12 months.

One-time cross-sectional survey; psychometric evaluation to confirm dimensions and create multi-item scales. Survey data were analyzed using VARCLUS and confirmatory factor analysis and internal consistency reliability testing.

**Results:**

Scales measuring five domains were confirmed: coordination within and between care teams, navigation (arranging appointments and visits), communication between specialist and primary care doctor, and communication between primary care doctor and specialist. Four of these demonstrated excellent internal consistency reliability. Mean scale scores indicated low levels of integration.

**Conclusion:**

These scales measuring integrated care capture meaningful domains of patients’ experiences of health care. The low levels of care integration reported by patients in the study sample suggest that these types of measures should be considered in ongoing evaluations of health system performance and improvement. Further research should examine whether differences in patient experience of integrated care are associated with differences in the processes and outcomes of care received.

**Electronic supplementary material:**

The online version of this article (doi:10.1186/s12913-016-1437-z) contains supplementary material, which is available to authorized users.

## Background

Patients often experience health care as fragmented and disjointed. An important health system goal is achieving “integrated care”— a sense of “cohesiveness and connectedness of the health care system” [1]. Much of the prior research on integrated care has considered integration as a structural property, focusing on geographic co-location of services and the organizational attributes of vertically integrated health delivery systems (e.g., Kaiser Permanente) or horizontally integrated entities (e.g., hospital chains) [2–4]. Various authors and organizations have investigated patients’ experience of integrated care, emphasizing functional aspects of integration such as care coordination and integration between health and social care [5]. Even the World Health Organization developed early thoughts on the subject and others have expanded on the proposed WHO framework [6, 7].

Several definitions have been proposed to capture a more holistic and patient-centered concept of integrated care. Singer and colleagues define integrated care as “patient care that is coordinated across professionals, facilities, and support systems; continuous over time and between visits; tailored to the patients’ needs and preferences; and based on shared responsibility between patient and caregivers for optimizing health” [4]. They proposed a conceptual framework consisting of seven domains: 1) coordination within care team, 2) coordination across care teams, 3) coordination between care teams and community resources, 4) continuous familiarity with patient over time, 5) continuous proactive and responsive action between visits, 6) patient-centered, and 7) shared responsibility. In a qualitative study exploring patients’ understanding and experiences of integrated care, we found that patients clearly perceive when integration and coordination are--or are not--happening in their experiences with the health care system, and that they highly value a sense of all members of the care team “being on the same page” [8]. The themes that emerged from these patient focus groups largely aligned with the seven domains of the Singer conceptual model.

Although our qualitative research lends support to the Singer conceptual framework, there are as yet no published, psychometrically sound survey instruments for systematically measuring all aspects of the patient experience of integrated care. Current instruments measure some, but not all of the concepts [9]. Many critical research questions about integrated care cannot be satisfactorily investigated without quantitative measures of patients’ experience of integrated care. For example, do patients receiving care from health care organizations with a high degree of vertical structural integration experience their care as more functionally integrated than patients receiving care in less tightly organized settings? Are minority and low socioeconomic status (SES) patients less likely than their counterparts to experience highly integrated care, and does that difference in experience partly explain disparities in medication adherence, preventive care services, diabetes control, avoidable hospitalizations, and other outcomes?

We developed and tested a self-report survey instrument to quantitatively measure patients’ experience of integrated care. In this article, we report the psychometric properties of several scales to measure patients’ experience of care integration.

## Methods

### Conceptual model

We used the Singer conceptual model to guide our instrument development, focusing on domains for which well-established self-report scales are unavailable. For this reason, we decided not to test new scales for the domains of patient-centeredness, continuity, and shared responsibility. Validated instruments exist for patient-centeredness, as well as continuity of care (which encompasses continuous familiarity) and shared decision-making (which encompasses shared responsibility) [10–15]. There is also extensive literature on some specific aspects of integration of specialty and primary care services, including co-location between behavioral healthcare providers and primary care providers [16–19]. We focused on the other four domains of the Singer model: coordination within care team, coordination across care teams, coordination between care teams and community resources, and continuous proactive and responsive action between visits. Based on findings from our focus groups, we added an additional domain of navigation using language used by patients. Patients in our focus groups frequently mentioned a desire for assistance in scheduling appointments and related care planning needs in complex health systems. Several questions were added that addressed needs to follow-up with clinics or other next steps for exploration.

### Evidence to select measures of integrated care

Through an extensive search of the literature, we started with a search of Pubmed MESH term “Delivery of Health Care, Integrated” to explode our search, including articles in the English language published between the dates of Jan. 1, 1985 – April 1, 2011. We included articles that were reviews, studies, and commentaries not limited to any specific disease, patient population, method or level of integration. This generated nearly 6000 articles, and based on titles we narrowed this list to less than 100 articles that appeared to include original research on patient experiences. We then narrowed the search further after reviewing article abstracts to identify publications that included patient survey measures of themes and elements of integrated care.

We reviewed existing survey instruments to identify questions that might capture aspects of the five domains we wanted to include in our integrated care instrument. Existing surveys that were most relevant to our study aims were the Consumer Assessment of Health Plans (CAHPS) survey [20], Commonwealth Fund International Patient Experience Survey [21], Tufts Medical Center Institute for Clinical Research and Health Policy Studies Ambulatory Care Experiences Survey [13, 22], the Patient Perceptions of Care Survey [23], and the Johns Hopkins Primary Care Assessment Tool [24]. We adapted items from these sources and developed new items to capture relevant concepts not adequately represented in these existing questionnaires.

### Cognitive interview pretesting

We performed cognitive interview pre-testing on 28 items that were either newly written or substantially modified from an existing survey item, and which we were concerned might be misinterpreted, misclassified, or be otherwise problematic, especially among individuals from lower SES groups. Probes were developed to determine whether respondents understood the intended meaning of specific words or phrases, whether similar questions were perceived as redundant, and whether questions were offensive; to identify the cognitive processes used in responding; and to describe examples from respondents’ experience. Individual cognitive interviews were conducted face-to-face with a convenience sample of 20 patients from clinics within San Francisco General Hospital. Patients were eligible if they were 40 years of age or older and had used the clinic at least two times within the past year. Participants were consented in person and were provided a gift card for their time. The UCSF Institutional Review Board approved this study protocol.

The pretest sample was mostly female (70 %), between the age of 40 to 55 (70 %), African-American (45 %), had a high school education or less (78 %), and uninsured (55 %). Interviews were conducted in English, with a translator used for two participants with limited English proficiency. As an example of a probe, for the item “How often did your regular doctor seem informed and up-to-date about the care you got from the specialist”, the interviewer queried, “What does the phrase informed and up-to-date mean to you?” Of the 28 items included in cognitive testing, 21 (75 %) presented either minor or major problems. Of these 21 questions, 11 (52 %) showed similar meaning to a prior question, 3 (14 %) had clarification problems, 11 (52 %) demonstrated response scale problems, and 7 (33 %) questions were repeated during interviews by the interviewer. Seven items were dropped, and several items were revised including refinement of response options and addition of new items to better capture concepts.

We also used the cognitive pretesting to assess the acceptability of our response scales, which consisted of respondents rating “how often” an item occurred in the past 12 months, using a scale ranging from *never* to *always*. This is the same response format used in the CAHPS survey, and our pretest sample of patients reported no difficulty in understanding this response scale [20].

### Fielded questionnaire items

The fielded questionnaire included 46 items addressing elements of integrated care. Additional file 1 lists these items and indicates the source of those items if they were adapted from existing questionnaires. All items used response options on a 6-level frequency scale regarding how often the integration experience occurred: 1 = never, 2 = almost never, 3 = sometimes, 4 = usually, 5 = almost always, or 6 = always. In addition to the items on experience of integrated care, the questionnaire included items on patient demographics, use of health care and general perceptions of the health care system. The survey was pretested with 10 participants before fielding the final survey.

### Survey sample

The surveys were administered by landline telephone interview in February 2012 to March 2012 using random digit dialing of households in the San Francisco Bay Area. Household members were eligible to participate in the survey if they were age 40 years or older and had used healthcare at least twice in the past 12 months, assessed in initial screening questions in the telephone interview. These eligibility criteria were used to preferentially recruit individuals who were active health care users and might have experiences in different facets of care integration. In our prior focus group work, we found that individuals who infrequently used health services could not describe the experience of integrated care. The concept is best described by those who have used multiple settings of healthcare. For this purpose, we estimated the likelihood based on the point estimates from the 2007 public dataset of the California Health Interview Survey [25]. On exploratory analyses of this survey data, we found that to recruit patients likely to have relatively high care coordination needs, we needed to include those who were 40 years of age or older, had one or more chronic condition (diabetes, hypertension, chronic lung disease, depression, chronic kidney disease, osteoarthritis, congestive heart failure, or mild cognitive impairment) and had at least two medical visits in the past 12 months. Each household was called back up to 6 times before abandoning the telephone number. The random digit dial approach precluded reaching those with only cell phones. Participants were consented orally at the time of survey administration and provided a $20 gift card for their time. The survey averaged 12 min in duration across participants.

### Analytical methods

Our analyses aimed to identify a set of multi-item scales to capture the different domains of integrated care that could be incorporated into a survey instrument for administration to a general population of adult patients. We began by examining item variability and missing data and then performed VARCLUS and confirmatory factor analyses, as well as scale-scale intercorrelations and internal consistency assessments, as part of an iterative process for determining final scales. Our analysis used SAS PROC VARCLUS, which is a SAS procedure to help a statistician quickly reduce the number of variables used to build a segmentation model. PROC VARCLUS clusters variables by finding groups of variables that are as correlated as possible among themselves and as uncorrelated as possible with variables in other clusters [26]. As input to PROC VARCLUS, we imputed a covariance matrix in SAS (Version 9.2). At this stage we made a decision to exclude items with a relatively high frequency of missing data. Missing data principally occurred because many respondents were not eligible to answer several item sets that were contingent on the individual receiving a specific service in the past 2 years or that were relatively low frequency events, such as a hospitalization or an emergency department visit. We excluded items from the analysis for which less than 40 % of the participants were eligible to answer. Our rationale for this decision was twofold. First, we did not want to excessively compromise the size of the study sample for psychometric testing. Second, we wanted to design survey scales that would be applicable to a wide population of patients and not just the relatively small proportion with the highest care needs.

The VARCLUS model suggested several clusters of items that were the basis for a final confirmatory factor analysis model. Because VARCLUS does not provide a means of assessing the empirical fit of the cluster solution, we used confirmatory factor analysis model to determine whether the VARCLUS solution provided a reasonable fit to the data.

For the confirmatory factor analysis, we used Mplus Version 5.21 with the same imputed covariance matrix as with PROC VARCLUS to test whether the items loaded on their theorized constructs, using 0.32 as the minimum acceptable value for factor loading, indicating that the item shares at least 10 % of its variation with the factor. Decisions on the adequacy of model fit to the hypothesized clusters were guided by the comparative fit index and the root mean square error of approximation), as well as modification indices. For all final scales, we calculated the internal-consistency reliability and examined the item-scale correlations corrected for overlap, to determine if these met a minimal criterion of being greater than 0.30. For internal consistency reliability, we used Cronbach’s alpha and considered an alpha reliability of 0.70 or greater to be acceptable. Values for final scales were computed as the mean of all scale items, with the minimum and maximum possible range of all item and scale scores being 1 and 6, respectively. Items worded in a negative fashion were reverse coded when computing scales, so that higher values for all items reflected better care integration.

### Preliminary test of construct validity

Rigorously testing construct validity was not one of the primary aims for this stage of scale development. However, we explored construct validity by testing the association of the scales with the insurance status of respondents. We hypothesized that patients with public insurance (Medicaid or Medicare) would report a less integrated experience of care than patients with private insurance. In California, because of the strong presence of managed care in the private health insurance market, including the vertically integrated Kaiser Permanente organization, patients with private insurance might be expected to experience care that is more proactively coordinated than adults with Medicaid and Medicare, which at the time of the survey were not predominantly managed care models in California. Our survey did not specifically ask about enrollment in a managed care plan, but did ask about the overall type of insurance.

## Results

Surveys were completed by 317 individuals, representing 64 % of contacted telephone numbers. The cooperation rate among those contacted and eligible was 87 %, and the overall response rate among those estimated to be eligible was 56 %. If eligibility rates were similar in participants who could not be reached, the response rate of eligible participants would be 63 % (12 % refusal rate; 87 % cooperation rate) [27]. The characteristics of respondents are shown in Table 1. The majority of respondents were female (62 %), of white race-ethnicity (80 %), and had graduated from college (61 %); 39 % were older than 65 years of age or older, and 21 % reported being in fair or poor health. Forty five percent had private insurance, 43 % had Medicare or Medicaid coverage, 12 % had other types of insurance and 2 % were uninsured.

**Table 1:** Characteristics of Survey Respondents (*N* = 317)

Characteristic	Categories	% (n)
Age in years	40–44	8.2 (26)
	45–50	14.2 (45)
	50–54	8.8 (28)
	55–64	30.1 (95)
	65–74	19.2 (61)
	>75	19.5 (62)
Gender	Female	62 (197)
Education	Less than high school	3.2 (10)
	High school	11.5 (36)
	Some college	23.9 (76)
	College	23.9 (76)
	More than 4 years college	37.5 (119)
Race	White	79.5 (252)
	Black	8.7 (28)
	Latino	8.2 (26)
	Other	3.6 (11)
Insurance	Medicaid only	2.9 (9)
	Medicare only	8.1 (25)
	Medicare and Medicaid	4.6 (15)
	Medicare and Private Insurance	26.3 (83)
	Private Insurance	45.1 (142)
	Other insurance	10.1 (31)
	Self-pay/no insurance	2.9 (12)
Health Status	Excellent	18.6 (59)
	Very good	31.1 (99)
	Good	29.7 (94)
	Fair	15.5 (49)
	Poor	5.1 (16)

Most respondents reported having a regular source of care (*N* = 296, 93 %), a blood test or other diagnostic test in the prior 12 months (*N* = 305, 96 %), an attempt to call their regular doctor’s office in the prior 12 months (*N* = 258, 81 %), and a visit to a specialist in the prior 2 years (*N* = 229, 72 %). Far fewer than half reported seeing a doctor at their regular place of care other than their own regular doctor in the prior 12 months (*N* = 110, 35 %), an attempt to email their regular doctor’s office in the prior 12 months (*N* = 99, 31 %), an overnight hospitalization in the prior 2 years (*N* = 57, 18 %), an emergency department visit in the prior 2 years (*N* = 110, 35 %), or use of community service organizations such as Meals on Wheels or wellness programs (*N* = 48, 15 %).

The number of participants responding to each of the integrated care items is shown in Additional file 1. As noted in the methods section, because a distinct minority of patients had recent experiences with seeing more than one doctor at their regular place of care, email communication with doctors, inpatient hospital care, and emergency department care, we excluded items addressing these care experiences from the factor analysis in order to test scales applicable to the majority of respondents. Table 2 lists the 26 items with sufficient numbers of respondents to be included in the VARCLUS, grouped by six hypothesized domains. Of the 26 items included in the VARCLUS, 21 loaded on five scales (Table 3). Items in one hypothesized domain (continuous/proactive action between visits) did not form a cluster, and one item in the general coordination domain did not cluster with its domain. The final five domains thus differed from the hypothesized domains slightly. The six items hypothesized to measure coordination between care teams distinguished themselves in terms of the direction of coordination - splitting into coordination between primary care doctors and specialists, and between specialists and primary care doctors. Items in the hypothesized “general coordination” and “coordination within care team” domain combined into one cluster we relabeled “coordination within and between care teams”.

**Table 2:** Integrated Care Items Included in VARCLUS Analysis

Hypothesized scale domain	Item number and content	Final scale disposition
General Coordination		
	6. In the last 12 months, when receiving care for a medical problem, how often did you receive conflicting or disagreeing information from different doctors?	Coordination within and between care teams
	7. In the last 12 months, how often did you have to repeat yourself, or explain your problem again, to different doctors?	Coordination within and between care teams
	8. In the last 12 months, how often were you confused because different doctors told you different things?	Coordination within and between care teams
	73. In the last 12 months, how often have you felt your time was wasted because your care was poorly organized or poorly coordinated?	Coordination within and between care teams
	74. In the last 12 months, how often have you had trouble getting your doctors coordinated?	Coordination within and between care teams
	78. In the last 12 months, how often did you know what the next step for your treatment would be?	Did not scale
Coordination within care team		
	29. In the last 12 months, how often did [your regular doctor/doctors] seem to know the important information about your medical history?	Coordination within and between care teams
	30. When you need care or treatment, how often [does your regular doctor or medical staff/do doctors or their medical staff] you see know important information about your medical history?	Coordination within and between care teams
Continuous and proactive and responsive action between visits		
	24. When you had blood tests, x-rays or other tests, how often did someone call or send you the results of your tests?	Did not scale
	26. In the last 12 months, how often were results from your recent tests available at your doctor’s office at the time of your appointment?	Did not scale
	16. In the last 12 months, when you phoned your regular doctor’s office during regular office hours, how often did you get an answer to your medical question that same day?	Did not scale
	18. In the last 12 months, when you phoned your regular doctor’s office, how often did you get an answer to your medical question as soon as you needed?	Did not scale
Coordination between care teams		
	42. In the last 12 months, how often did you feel the specialists you saw had all the information they needed from your medical history?	Communication between primary care doctor and specialist
	43. In the last 12 months, when you saw a specialist, how often were you given enough information about why you were there by your [regular doctor/doctors]?	Communication between primary care doctor and specialist
	44. In the last 12 months, when seeing the specialists how often did he or she have enough information from your [regular doctor/doctors]?	Communication between primary care doctor and specialist
	5. In the last 12 months, after you saw the specialists how often did your [regular doctor/doctors] know what happened at your visit with the specialist?	Communication between specialist and primary care doctor
	46. In the last 12 months, after you saw the specialists how often did your [regular doctor/doctors] seem informed and up-to-date about the care you got from the specialists?	Communication between specialist and primary care doctor
	48. In the last 12 months, after you saw a specialist, how often did your [regular doctor/doctors] talk with you about what happened at the visit?	Communication between specialist and primary care doctor
Coordination with Community Resources		
	73. When you need care or treatment, how often [does your regular doctor or medical staff/do doctors or their medical staff] discuss with you different places you could go to get help with that problem or concern?	Coordination with community resources
	67. How often [does your regular doctor/do your doctors] help you find additional health related services, if you so choose?	Coordination with community resources
Navigation		
	34. In the past 12 months, how often [does your regular doctor or staff/do doctors or their staff] help schedule your appointments, referrals or tests?	Navigation-Care team arranged appointments and visits
	68. If you needed another visit with your regular doctor, how often did the staff do everything they could to make necessary arrangements or appointments?	Navigation-Care team arranged appointments and visits
	69. If you needed another visit with another doctor, how often did the staff do everything they could to make necessary arrangements or appointments?	Navigation-Care team arranged appointments and visits
	70. If you needed lab or radiology tests scheduled, how often did the staff do everything they could to make necessary arrangements or appointments?	Navigation-Care team arranged appointments and visits
	71. If you needed another visit with your regular or another doctor, how often did the staff do everything they could to make necessary arrangements when you thought you needed it?	Navigation-Care team arranged appointments and visits
	72. If you needed another visit with your regular or another doctor, how often did the staff do everything they could to make necessary arrangements at your preferred location?	Navigation-Care team arranged appointments and visits

Response categories for each item: 1 = never, 2 = almost never, 3 = sometimes, 4 = usually, 5 = almost always, 6 = always

**Table 3:** Results of Standardized Confirmatory Factor Analysis Loadings

Scale	Standardized loading	Standard error
Coordination within and between care teams		
6. Received conflicting information	.616	.048
7. Needed to repeat self	.679	.043
8. Confused because of conflicting information	.619	.048
29. Doctors know medical history	.671	.043
30. Staff knows medical history	.663	.037
73. Time wasted because of poor coordination/organization of care	.667	.042
74. Trouble getting doctors coordinated.	.724	.038
Navigation: Care team arranged appointments and visits		
34. Help scheduling follow-up appointments	.561	.051
68. Made arrangements for visits with regular doctor	.671	.046
69. Made arrangements for visits with other doctors	.837	.026
70. Made arrangements for lab or other tests	.796	.031
71. Made arrangements in a timely manner (when needed)	.857	.024
72. Made arrangements at preferred location (when needed)	.767	.033
Communication between specialist and primary care doctor		
45. Specialist gave information to primary care doctor	.926	.017
46. Primary care doctor seemed informed after specialist visit	.959	.016
48. Primary care doctor talked to patient about specialist visit	.612	.045
Coordination with community resources		
33. Discussed different places for help/treatment	.526	.069
67. Help with other health resources	.700	.074
Communication between primary care doctor and specialist		
42. Specialist had all basic information	.774	.036
43. Patient knew why needed specialist visit	.667	.045
44. Specialist had enough information from primary care doctor	.899	.028

For the confirmatory factor analysis, we therefore tested the 5-domain structure identified in the final VARCLUS. All 21 items loaded on their theorized constructs, using 0.32 as the minimum acceptable value for factor loading. The comparative fit index (CFI) was 0.889 and the root mean square error of approximation (RMSEA) was 0.080.

The “coordination within and between care teams” scale comprised 7 items (received conflicting information, needed to repeat self, confused because of conflicting information, doctors know medical history, staff knows medical history, time wasted because of poor coordination/organization of care, trouble getting doctors coordinated). The “navigation” scale comprised 6 items (help scheduling follow-up appointments, made arrangements for visits with regular doctor, made arrangements for visits with other doctors, made arrangements for lab and other tests, made arrangements in a timely manner, made arrangements at preferred location). The “communication between specialist and primary care doctor” scale comprised 3 items (specialists gave information to primary doctor, primary care doctor seemed informed after specialist visit, and primary care doctor talked to patient about specialist visit). Only two items loaded on the “coordination with community resources” dimension (discussed different places for help/treatment, help with other resources). The “communication between primary care doctor and specialist” scale also had three items (specialist had all basic information, patient knew why needed specialist visit, specialist had enough information from primary care doctor).

Table 4 shows the summary statistics for the five scales including number with complete data, unstandardized alpha, the range of item-scale correlations corrected for overlap, the mean and standard deviation, and the interquartile range. Internal-consistency reliabilities ranged from 0.55 to 0.87; alphas were greater than 0.70 for four of the five scales, but did not meet our minimum criteria of 0.70 for the “coordination with community resources” scale. Mean scale scores were relatively low, ranging from 1.9 (coordination within and between care teams) to 2.8 (communication between specialist and primary care doctor). These means represent ratings of integration falling in the “almost never” or “sometimes” occurring range.

**Table 4:** Reliability, Sample Size, Item-scale correlations, and Descriptive Statistics (*N* = MAX 229)

Scale^a^	No. of items	Number	Item numbers	Unstand-ardized Alpha	Range of item-scale correlations	Mean (SD)	Inter- quartile range
Coordination within and between care teams	7	229	6 (R), 7 (R), 8 (R), 29, 30, 73 (R), 74 (R)	0.85	0.57–0.66	1.9 (0.86)	1.14–2.43
Navigation: arranged appointments and visits	6	228	34, 68–72	0.87	0.52–0 .80	2.1 (1.33)	1.0–2.83
Communication between specialist and primary care doctor	3	224	45, 46, 48	0.86	0.59–0 .83	2.8 (1.72)	1.0–4.0
Coordination with community resources	2	223	33, 67	0.55	0.38	2.7 (1.54)	1.0–4.0
Communication between primary care doctor and specialist	3	228	42–44	0.80	0.58–0.75	2.0 (1.32)	1.0–2.67

*R* reversed scored items

*SD* standard deviation

^a^All scales are scored so that a higher score is more integrated care, with possible range of 1–6

Table 5 presents preliminary construct validity results. As hypothesized, there was a consistent trend across all scales for the mean ratings of integration to be higher among patients with private insurance than with public insurance, with the difference achieving statistical significance for “coordination with community resources”.

**Table 5:** Mean Scores for Integrated Care Scales, According to Insurance Type (Public vs. Private)

Scale^a^	Mean (SD)		*P*-value
	Public	Private	
Number of respondents	175	142	
Coordination within and between care teams	1.84 (0.83)	2.04 (0.92)	0.10
Navigation: arranged appointments and visits	2.04 (1.31)	2.26 (1.39)	0.30
Communication between specialist and primary care doctor	2.75 (1.72)	2.98 (1.76)	0.40
Coordination with community resources	2.57 (1.45)	3.04 (1.72)	0.04
Communication between primary care doctor and specialist	1.93 (1.31)	2.19 (1.36)	0.20

*SD* standard deviation

^a^All scales are scored so that a higher score is more integrated care, with a possible range of 1–6 (never to always)

## Discussion

We succeeded in developing five scales to measure several dimensions of patients’ experience of care integration that have acceptable psychometric and factor loading properties. Four of the scales demonstrate excellent internal consistency reliability; one scale (coordination with community resources) had inadequate internal consistency, possibly because it only consisted of two items. Nonetheless, this scale demonstrated significant differences between patients with private and public insurance indicating some degree of validity, suggesting that the internal-consistency statistic may be an incomplete estimate of its reliability. All these scales measure dimensions of integrated care that generally align with the conceptual model developed by Singer and colleagues and were endorsed by patients in our prior qualitative study of patients’ experience of care integration [4, 8]. The “coordination within and between care teams” scale appears to represent the patient’s overall sense of integration, including the adequacy of informational continuity and care planning when multiple doctors and other personnel participate in a patient’s care. We hypothesized that all items addressing communication between the patient’s primary care physician and specialists participating in the patient’s care would fall into a single construct. However, the VARCLUS indicated that there were two distinct constructs in this domain, distinguished by the direction of the communication. One scale included items on communication to the specialist from the patient’s primary care physician and others involved in the patient’s care, and the other included items on communication from the specialist back to the primary care physician. The “navigation” scale straddles two of the domains in the Singer conceptual model: patient centered and continuous proactive and responsive action between visits. We labeled this scale “navigation” because it captured a logistical aspect of care integration involving patients getting assistance with scheduling services and tests in a patient-centered, coordinated manner.

The low mean scores on all scales suggest that the patients we studied are experiencing care that is anything but highly integrated from their point of view. The highest mean score - 2.8 for “communication between specialist and primary care doctor” - falls below a value of 3 which would represent an experience of “sometimes” experiencing care in that domain as being well integrated. We used response categories that assessed the frequency with which various features of care were experienced (a never-to-always scale) rather than assessing their ratings of the quality of those experiences (e.g., a poor-to-excellent scale) because of our focus on what occurred rather than patients’ evaluation of their experience. We believe that most health systems would consider it inadequate for the average patient to experience care being integrated only some of the time and not on a consistent basis.

We hoped to measure additional domains of care integration, such as coordination involving hospital and emergency department visits. However, most individuals in the community do not have hospitalizations or emergency department visits within the prior 1 to 2 years, making it difficult to include items addressing these care components in scales of overall care integration applicable to the general population. Even using inclusion criteria which were designed to target patients with greater health care needs, only about one-third or fewer of the patients in our sample had recent encounters in several of the health care areas we considered relevant for understanding care integration. We could have created separate scales focusing only on these less frequent types of care experiences, but our goal for this first generation of scale development was to emphasize scales applicable to a large segment of the population who are active users of health care.

Our study has several limitations. Our questionnaire items should not be considered an exhaustive set of items for measuring important aspects of the experience of care integration, and there is undoubtedly opportunity to expand upon and further refine scales on care integration domains. We surveyed patients in one geographic region of California, and their experiences and responses may not be representative of individuals living in other regions. Survey participants were age 40 years or older; responses may not reflect pediatric or geriatric populations’ experiences with care. We did not design our questionnaire and sample size to perform a rigorous assessment of construct validity or test its use in a real-world setting. In real-world situations, patients would not be consented to participate in the survey for research purposes or receive an incentive, although patients likely would answer questions such as these outside of the clinical encounter, on a phone call and not at random. While the trends for an association between type of insurance and patient reports of care integration are suggestive, further study will be needed to more systematically examine the construct validity of these scales.

Although the response rate was relatively high for a random digit dial telephone survey, there is always a possibility that response bias may have influenced our findings. Our sample also reflects a higher percentage of those with a college education, white and older than the Census data [28]. However, similar findings have resulted from other household telephone based surveys. Younger people, minorities, and lower SES are more likely to be found in cell-phone-only homes and may bias the results of a landline based survey [29]. The major limitation of our study is that we were not able to test further the construct validity of the scales or test predictive validity. We also note that some domains used less frequently are difficult to assess. Coordination with community resources and communication between providers may be highly situational. For this reason additional measures from existing validated surveys may be added to supplement this scale. Future research should pursue validation studies, for example by investigating if low scores on these self-report scales of care integration predict outcomes such as patients changing their regular source of care, duplication of diagnostic tests, preventable hospitalizations, and adverse medication events.

Our study has several policy implications. Foremost is the suggestion that health systems should engage patients in order to understand their experiences as systems attempt to deliver care that is more seamlessly coordinated and patient-centered. Much of the effort in care integration in the US has focused on structurally integrating various delivery components under a more corporate model [30], as well as placing great hope in health information technology as a solution to poorly coordinated care [31, 32]. Although we did not ascertain which patients in our study may have received care in more structurally integrated systems such as Kaiser Permanente or whether their caregivers used electronic health records, the California health care market is distinguished by a high prevalence of managed care, structurally integrated delivery systems, and adoption of electronic health records [33]. The relatively low ratings that most respondents gave to their experience of care integration suggests that systems should carefully assess whether strategies for promoting integration are making patients experience care as more functionally integrated and enhancing patients’ perceptions that everyone involved in their care is “on the same team [8]”. Engaging patients in advisory councils and as partners on care redesign teams is one approach that may help health systems to tune into the patient voice and patient insights about what makes care truly patient-centered and well-coordinated [34]. If further validated, health systems may find these patient-reported scales of care integration to be helpful in informing them about current strengths and weaknesses in these aspects of care coordination. These scales also may provide a tool for assessing whether interventions to improve health system performance are producing improvements in patients’ experience of care integration.

Future implications of having five scales that measure several dimensions of patients’ experience of care integration suggest that in broader settings we may be able to further explore which parts of integrated care matter most. After the scales are further evaluated and tested in real-world settings, we may have further insights about how the instrument may be used and linked to outcomes. Expanding this evidence base may require additional work and inquiry.

Several areas of future research are important to note. It is clear that a complete understanding of integrated care will require a combination of perspectives from patients, clinicians, and administrators. Even then, several survey instruments may need to be combined to gauge the appropriate measurement of integration. Future studies may need to assess how to combine scales across perspectives and instruments.

## Conclusions

In conclusion, we have developed several patient self-report scales to measure important domains of the patient experience of integrated care. These scales may prove to be useful to efforts that more systematically evaluating health care reforms. The scales may also provide a starting point for researchers seeking to study, evaluate and analyze delivery models that strive to promote greater coordination of health services. The end goal is to better understand common elements that can provide patients with a more seamless experience with care and promote optimal health outcomes.

## Abbreviations

CAHPS, consumer assessment of health plans; CFI, comparative fit index; MeSH, medical subject headings; RMSEA, root mean square error of approximation; UCSF, University of California, San Francisco; WHO, World Health Organization.

## Additional file

Additional file 1: Survey Items. (DOCX 30 kb)
