# Supplementary material for: Development of a survey instrument to measure patient experience of integrated care
Source: BMC Health Serv Res. 2016 Jun 1;16:193. doi: 10.1186/s12913-016-1437-z (PMC4890282; doi:10.1186/s12913-016-1437-z)
Supplement: Additional file 1: — Survey Items. (DOCX 30 kb) [file 12913_2016_1437_MOESM1_ESM.docx]

**^Additional file 1: Survey Items^**

All 46 Items Hypothesized to Measure Aspects of Integrated Care and Included in Final Survey, with Number of Respondents to Each Item and Source of Item if Adapted From an Existing Questionnaire.

Instructions for the Final Survey: When answering the survey questions, please think only about your own health care. Throughout the survey, we will refer to the place you usually go to for most of your medical care as "your regular doctor's office.

Response choices were rated “how often” an item was true for them with endpoints labeled 0 = *never* and 5 = *always*.”

| *Hypothesized Scale Domain* | *Item* | *Number of study participants responding to item (total N= 317)* | *Original Source of Item, if Adapted from Existing Questionnaire* |
| --- | --- | --- | --- |
| General Coordination |  |  |  |
|  | 6. In the last 12 months, when receiving care for a medical problem, how often did you receive conflicting or disagreeing information from different doctors? | 309 | Commonwealth |
|  | 7. In the last 12 months, how often did you have to repeat yourself, or explain your problem again, to different doctors? | 313 | New |
|  | 8. In the last 12 months, how often were you confused because different doctors told you different things? | 309 | PPC |
|  | 73. In the last 12 months, how often have you felt your time was wasted because your care was poorly organized or poorly coordinated? | 314 | Commonwealth |
|  | 74. In the last 12 months, how often have you had trouble getting your doctors coordinated? | 308 | New |
|  | 78. In the last 12 months, how often did you know what the next step for your treatment would be? | 294 | PPC |
| Coordination within care team |  |  |  |
|  | 29. In the last 12 months, how often did [your regular doctor/doctors] seem to know the important information about your medical history? | 309 | CAHPS |
|  | 30. When you need care or treatment, how often [does your regular doctor or medical staff/do doctors or their medical staff] you see know important information about your medical history? | 309 | Commonwealth |
|  | *Items 36-39 asked of the 110 respondents who reported seeing any doctors at their regular place other than their regular doctor in the past 12 months…* |  |  |
|  | 36. How often were the other doctors who cared for you familiar with your medical history? | 108 | New |
|  | 37. How often did you feel that the doctors talked to one another? | 102 | New |
|  | 38. How often did you feel that the other doctors were able to see your medical history and symptoms in the medical records? | 109 | PPC |
|  | 39. How often did you feel that the other doctors and nurses you saw in your regular doctor's office had all the information they needed to correctly diagnose and treat your health problem? | 107 | CAHPS |
| Continuous and proactive and responsive action between visits |  |  |  |
|  | *Items 24 and 26 asked of the 305 respondents who reported having blood tests, x-rays or other tests in the past 12 months…* |  |  |
|  | 24. When you had blood tests, x-rays or other tests, how often did someone call or send you the results of your tests? | 289 | New |
|  | 26. In the last 12 months, how often were results from your recent tests available at your doctor's office at the time of your appointment? | 290 | New |
|  | *Items 16 and 18 asked of the 258 respondents who reported trying to phone their regular place of care in the past 12 months…* |  |  |
|  | 16. In the last 12 months, when you phoned your regular doctor's office during regular office hours, how often did you get an answer to your medical question that same day? | 244 | CAHPS |
|  | 18. In the last 12 months, when you phoned your regular doctor's office, how often did you get an answer to your medical question as soon as you needed? | 242 | New |
|  | *Items 20 and 21 asked of the 99 respondents who reported trying to email their regular place of care in past 12 months…* |  |  |
|  | 20. In the last 12 months, when you e-mailed your regular doctor's office, how often did you get an answer to your medical question as soon as you needed? | 98 | CAHPS |
|  | 21. In the last 12 months, when you e-mailed your regular doctor's office, how often did the reply come from someone who could answer your question? | 96 | New |
| Coordination between care teams |  |  |  |
|  | *Items 42-46 and 48 asked of the 229 respondents who reported seeing a specialist doctor in past 2 years…* |  |  |
|  | 42. In the last 12 months, how often did you feel the specialists you saw had all the information they needed from your medical history? | 224 | PPC |
|  | 43. In the last 12 months, when you saw a specialist, how often were you given enough information about why you were there by your [regular doctor/doctors]? | 216 | New |
|  | 44. In the last 12 months, when seeing the specialists how often did he or she have enough information from your [regular doctor/doctors]? | 215 | Commonwealth |
|  | 45. In the last 12 months, after you saw the specialists how often did your [regular doctor/doctors] know what happened at your visit with the specialist? | 203 | PCAT |
|  | 46. In the last 12 months, after you saw the specialists how often did your [regular doctor/doctors] seem informed and up-to-date about the care you got from the specialists? | 209 | ACES |
|  | 48. In the last 12 months, after you saw a specialist, how often did your [regular doctor/doctors] talk with you about what happened at the visit? | 216 | PCAT |
|  | *Items 50-53 asked of the 57 respondents who reported being admitted overnight to a hospital in past 2 years…* |  |  |
|  | 50. When you left the hospital, how confident were you of the purpose of taking each of your medications? | 56 | Commonwealth |
|  | 51. When you left the hospital, did the hospital make arrangements or make sure you had follow-up visits with a doctor? | 57 | Commonwealth |
|  | 52. After you left the hospital, how confident were you that your [regular doctor/doctors] seemed informed and up-to-date about the care you received in the hospital? | 58 | Commonwealth |
|  | 53. After you left the hospital, how confident were you that your [regular doctor/doctors] had access to all of your information about your hospital stay? | 57 | New |
|  | *Items 56-59 asked of the 110 respondents who reported using a hospital emergency department in the last 2 years…* |  |  |
|  | 56. The last time you went to the emergency room was it for a condition that you thought could have been treated by the doctors or staff at the place where you usually get medical care if they had been available? | 97 | Commonwealth |
|  | 57. The last time you went to the emergency room did you feel the doctors asked you questions they should have asked your regular doctor instead of you? | 102 | New |
|  | 58. After your visit in the hospital emergency room, how confident were you that the doctors or staff at the place where you usually get medical care seemed informed and up-to-date about the care you had received in the hospital emergency room? | 102 | Commonwealth |
|  | 59. After your visit, how confident were you that your regular doctor had access to all of your information about your visit to the emergency room? | 98 | Commonwealth |
| Coordination with Community Resources |  |  |  |
|  | 33. When you need care or treatment, how often [does your regular doctor or medical staff/do doctors or their medical staff] discuss with you different places you could go to get help with that problem or concern? | 291 | PCAT |
|  | 67. How often [does your regular doctor/do your doctors] help you find additional health related services, if you so choose? | 286 | FCC |
|  | *Items 76-77 asked of the 48 respondents who reported needing to use outside organizations to help you with their care such as Meals on Wheels and community wellness programs…* |  |  |
|  | 76. How often does your regular doctor include community based services for you or your family in your medical record? | 45 | New |
|  | 77. How often does your regular doctor have a staff person that helps your family connect with needed community-based services? | 47 | New |
| Navigation |  |  |  |
|  | 34. In the past 12 months, how often [does your regular doctor or staff/do doctors or their staff] help schedule your appointments, referrals or tests? | 302 | New |
|  | 68. If you needed another visit you’re your regular doctor, how often did the staff do everything they could to make necessary arrangements or appointments? | 255 | PCC |
|  | 69. If you needed another visit with another doctor, how often did the staff do everything they could to make necessary arrangements or appointments? | 284 | PCC |
|  | 70. If you needed lab or radiology tests scheduled, how often did the staff do everything they could to make necessary arrangements or appointments? | 290 | New |
|  | 71. If you needed another visit with your regular or another doctor, how often did the staff do everything they could to make necessary arrangements when you thought you needed it? | 300 | New |
|  | 72. If you needed another visit with your regular or another doctor, how often did the staff do everything they could to make necessary arrangements at your preferred location? | 296 | New |

Response categories for each item: 1=never, 2=almost never, 3=sometimes, 4=usually, 5=almost always, 6=always.

**The authors are happy to provide the full survey upon request, including questions that did not scale.**

*Sources of items adapted from existing questionnaire instruments (wording of derived items was often modified for use in integrated care survey instrument):*

CAHPS: Consumer Assessment of Health Plan Survey; Adult Primary Care Questionnaire 1.0 and Supplemental Items updated Oct. 8, 2009

Commonwealth: Commonwealth International Health Project 2010

ACES: Ambulatory Care Experiences Survey; Tufts Medical Center: Institute for Clinical Research and Health Policy Studies Web site.

Available at: http://160.109.101.132/icrhps/resprog/thi/aces_publist.asp Accessed: 21 September 2010.

PPC: Patient Perceptions of Care: Borowsky SJ, Nelson DB, Fortney JC, et al. VA Community-Based Outpatient Clinics. Performance measures based on patient perceptions of care. Med Care 2002;40(7):578-86

PCAT: Primary Care Assessment Tool. Shi L, Starfield B, Xu J. Validating the Adult Primary Care Assessment Tool. J Fam Pract 2001;50:161W-175W.

FCC: Family Centered Care Self-Assessment Tool. Available at <http://www.familyvoices.org/admin/work_family_centered/files/fcca_FamilyTool.pdf>

New: item newly drafted for integrated care survey
